# Cell-Laden Hydrogel as a Clinical-Relevant 3D Model for Analyzing Neuroblastoma Growth, Immunophenotype, and Susceptibility to Therapies

**DOI:** 10.3389/fimmu.2019.01876

**Published:** 2019-08-09

**Authors:** Alessandra Marrella, Alessandra Dondero, Maurizio Aiello, Beatrice Casu, Daniel Olive, Stefano Regis, Cristina Bottino, Daniela Pende, Raffaella Meazza, Guido Caluori, Roberta Castriconi, Silvia Scaglione

**Affiliations:** ^1^CNR—IEIIT Institute, National Research Council of Italy, Genoa, Italy; ^2^Department of Experimental Medicine, University of Genoa, Genoa, Italy; ^3^React4life S.r.l., Genoa, Italy; ^4^Tumor Immunology Team, IBISA Immunomonitoring Platform, Cancer Research Center of Marseille, INSERM U1068, CNRS U7258, Institut Paoli-Calmettes, Aix-Marseille University, Marseille, France; ^5^Laboratory of Clinical and Experimental Immunology, IRCCS Giannina Gaslini, Genoa, Italy; ^6^Laboratorio di Immunologia, IRCCS Ospedale Policlinico San Martino, Genoa, Italy; ^7^FNUSA-ICRC, Interventional Cardiac Electrophysiology, Brno, Czechia; ^8^Nanobiotechnology, CEITEC Masaryk University, Brno, Czechia; ^9^Centre of Excellence for Biomedical Research, CEBR, University of Genoa, Genoa, Italy

**Keywords:** 3D cancer model, neuroblastoma, NK cells, PVR, KIR_S_/HLA-I, PD-Ls, B7-H3 and IFN-γ, immunotherapy

## Abstract

High risk Neuroblastoma (NB) includes aggressive, metastatic solid tumors of childhood. The survival rate improved only modestly, despite the use of combination therapies including novel immunotherapies based on the antibody-mediated targeting of tumor-associated surface ligands. Treatment failures may be due to the lack of adequate *in vitro* models for studying, in a given patient, the efficacy of potential therapeutics, including those aimed to enhance anti-tumor immune responses. We here propose a 3D alginate-based hydrogel as extracellular microenvironment to evaluate the effects of the three-dimensionality on biological and immunological properties of NB cells. NB cell lines grown within the 3D alginate spheres presented spheroid morphology, optimal survival, and proliferation capabilities, and a reduced sensitivity to the cytotoxic effect of imatinib mesylate. 3D cultured NB cells were also evaluated for the constitutive and IFN-γ-induced expression of surface molecules capable of tuning the anti-tumor activity of NK cells including immune checkpoint ligands. In particular, IFN-γ induced de novo expression of high amounts of HLA-I molecules, which protected NB cells from the attack mediated by KIR/KIR-L matched NK cells. Moreover, in the 3D alginate spheres, the cytokine increased the expression of the immune checkpoint ligands PD-Ls and B7-H3 while virtually abrogating that of PVR, a ligand of DNAM-1 activating receptor, whose expression correlates with high susceptibility to NK-mediated killing. Our 3D model highlighted molecular features that more closely resemble the immunophenotypic variants occurring *in vivo* and not fully appreciated in classical 2D culture conditions. Thus, based on our results, 3D alginate-based hydrogels might represent a clinical-relevant cell culture platform where to test the efficacy of personalized therapeutic approaches aimed to optimize the current and innovative immune based therapies in a very systematic and reliable way.

## Introduction

Neuroblastoma (NB) is a pediatric cancer accounting for 6% of all children malignancies (1, 2). It represents a worldwide emergency since it is responsible for up to 10% of mortality in childhood cancer. In particular, high-risk NBs are extremely aggressive as they present resistance to therapy and relapses, mainly due to recurrence of bone marrow metastasis. Thus, an unmet therapeutic need exists that prompts the scientific community to identify innovative therapeutic strategies, which may include immunotherapies based on the enhancement of Natural Killer (NK) lymphocytes activity.

NK cells are the cytolytic members of the Innate Lymphoid Cells family (ILC) (3, 4). The capability of NK cells to attack abnormal targets such as tumors, while sparing healthy cells, depends on a balance between inhibitory and activating signals. The strongest inhibitory signals are mediated by the heterodimer CD94/NKG2A and by Killer Ig-like receptors (KIRs), clonally distributed surface receptors interacting with specific HLA class I (HLA-I) allotypes, referred as KIR-ligands (KIR-L). These inhibitory receptors tune the effector functions of NK cells and regulate their cross-talk with other immune cells such as Dendritic Cells (5) or Macrophages (6), a process that lead to the amplification of innate and adaptive immune responses. Moreover, during NK cell maturation, the engagement of HLA-I-specific receptors is required for NK cell “licensing,” a complex educational program that allow NK cells to acquire a fully functional phenotype getting the “license to kill” (7). Indeed, mature “licensed” NK cells are characterized by high cytolytic potential and by the expression of a plethora of functional activating receptors and co-receptors. These include NKp46 (8), NKp30 (9), NKp44 (10) (collectively named Natural Cytotoxicity Receptors, NCRs), NKG2D (11), and DNAM-1 (12) that, interacting with specific ligands on tumor targets, stimulate NK cells killing, and cytokines release (13). In particular, the interaction of DNAM-1 with its ligand PVR (polio virus receptor, CD155) (14) plays a pivotal role in cell-to-cell adhesion (15) and in immune synapses formation. It has been shown to be crucial for killing of *ex-vivo* derived tumors including NB that do not express HLA-I molecules at the cell surface (16–21).

The expression of HLA-I surface molecule in tumor cells can impair the NK cell-mediated attack through the engagement of the specific inhibitory receptors. Moreover, tumors can evade immune responses via the exploitation of the “immune checkpoints,” inhibitory pathways that physiologically maintain self-tolerance and limit the duration and amplitude of immune responses. These include the PD-L1 and PD-L2 ligands recognized by the Programmed cell death 1 (PD-1) receptor (22–24), and the B7-H3 orphan ligand interacting with a still unknown receptor (25, 26). PD-Ls and B7-H3 are expressed/upregulated by tumors and act as shields protecting cancer cells from the NK (and T) cells attack (27, 28). Notably, IFN-γ, which is released by NK (and Th1) cells during immune responses, is able to increase the expression of HLA-I, and PD-L1 molecules in different tumor cells including NB (23, 29–31).

Novel therapeutic approaches in high-risk NB patients consider the enhancement of immune responses through the disruption of the PD-1/PD-Ls and/or B7-H3R/B7-H3 axes (27, 28). An additional possibility is targeting B7-H3, which can be achieved by using antibodies (32) or B7-H3-CAR engineered T (33, 34) or NK cells (27). In this context, B7-H3 has not been detected in most normal tissues including the nervous system (35, 36).

Based on experimental evidences obtained from *in vitro* and mice animal models, combined therapies have been designed; however, the patients' survival rate was poorly improved. Clinical failure may be due to several reasons including the inadequacy of the simplistic pre-clinical *in vitro* and *in vivo* animal tumor models. Conventional 2D cultures do not allow the persistence of NB cells isolated from patients, hampering the evaluation of the responses to therapy in a single patient, as required by personalized medicine that takes into account the great individual biological variability.

Therefore, there is an exigency to develop novel *in vitro* 3D models characterized by more reliable dimensional context and higher degree of physiological relevance and suitable for approaching personalized immunotherapies. To meet this need, bioengineering of the tumor microenvironment is beneficial, and biomaterials play a role in this endeavor. Among these, bioengineered 3D hydrogels can provide a link between *in vitro* and *in vivo* systems, since they well resemble the special characteristics of the tumor microenvironment, including tunable stiffness and ductility modulations, programmed degradability, cancer-specific biomarker responsibility, and other properties (37, 38).

Among them, seaweed-derived alginate is an inert polymer, lacking the native bonds, which are usually responsible of the interactions with mammalian cells, differently from natural polymers like collagen or laminin (39–41). For this reason, alginate-based materials allow to better isolate and distinguish the contribution of the physico-structural properties of the substrates on cell fate, respect to chemically bioactive materials (42, 43).

The architecture, stiffness, and composition of the extracellular matrix (ECM) could in fact affect cell migration, invasion, and proliferation in human cancers as well as the cellular immune-phenotype and the sensitivity to anticancer drugs and immunotherapeutic approaches. Therefore, we here developed and validated a biomimetic culture platform based on cell-laden alginate-based spheroids as promising and reliable NB 3D tumor model. In particular, a characterization of cell viability, proliferation, drug sensitivity and ligand repertoire has been carried out, by using two prototypic human NB cell lines. Compared with conventional 2D cell monolayer cultures, NB cells grown within the 3D alginate hydrogels present a spheroid morphology, resembling *in vivo* organization, a decreased sensitivity to the cytotoxic effect of imatinib mesylate and revealed an interestingly and previously unappreciated, constitutive and IFN-γ induced, immune-phenotype.

## Materials and Methods

### NB Cell Lines and Cell Culture

The NB cell line HTLA-230 was provided by Dr. E. Bogenmann (Children's Hospital Los Angeles, Los Angeles, CA) (44); the SH-SY5Y cell line was purchased from Banca Biologica and Cell Factory (IRCCS Azienda Ospedaliera Universitaria San Martino-IST, Genoa, Italy). NB cell lines were cultured in the presence of complete medium composed of RPMI 1640 medium supplemented with 10% heat-inactivated FCS (Biochrom, Berlin, Germany), 50 mg/ml streptomycin, 50 mg/ml penicillin (Sigma-Aldrich), and 2 mM glutamine (EuroClone), either in the absence or in the presence of INF-γ (100 ng/ml). NB cell lines were periodically checked for *MYCN* gene amplification by fluorescence *in situ* hybridization analysis and for morphology, proliferation rate, and mycoplasma contamination, after thawing and within four passages in culture. KIR-L were attributed analyzing the HLA-I typing, using the website http://www.ebi.ac.uk/ipd/kir/ligand.html. Cells were used to realize 3D tumor model (see below) and conventional 2D cultures (10,000 cells/well in in 24-well flat-bottom plates).

### 3D Tumor Model Formation and Culture

Alginate powder (Manugel GMB, FMC Biopolymer) was dissolved in physiologic solution at the concentration of 1% w/v and the solution was filtered through a 0.2 mm membrane under sterile conditions. HTLA-230 and SH-SY5Y were detached from plastic tissue culture flasks by using phosphate-buffered saline containing 5 mM EDTA and resuspended at a density of 4 × 10^6^ cells/mL in DMEM supplemented with 10% FBS and 1% penicillin/streptomycin. Afterward, the NB cells suspension was mixed with the sterile alginate solution (1/1 V/V) to obtain a final Alg concentrations of 0.5% w/V and a cellular density of 2 × 10^6^ cells/mL. The NB cells/alginate suspensions were dripped by 21-gauge needle into a 0.5 M CaCl_2_ gelling bath and kept for 10 min at 37°C to form alginate spheres. After washing the spheres with DI water to remove the excess of Ca, the NB cell-laden alginate spheres were cultured in 96 multi-wells up to 1 week in DMEM supplemented with 10% FBS, 1% penicillin/streptomycin and CaCl_2_ (5 mM). They were incubated in a humidified environment (5% CO2) at 37°C and the medium was changed after 3 days (Patent n° 102018000005459; Owner: React4life S.r.l.).

### Dimensional Characterization of the NB Cell-Laden Alginate Spheres

The morphology of NB cell-laden alginate spheres was monitored up to 1 week of culture. After 1, 3 and 7 days of culture NB-alginate spheres were fixed in 4% paraformaldehyde and stained with DAPI fluorescent nuclear dye to observe NB cells nuclei. Imaging was performed with a fluorescence microscope (Nikon H550L). Images obtained by fluorescence microscopy were analyzed through Image J software, in order to quantify the eventual temporal change of the morphological features of NB cell-laden alginate spheres. In particular, the following morphological parameters were measured: diameter; area; circularity, defined as 4π Area/ Perimeter^2^; and axis ratio, defined as Major Axis/Minor axis.

### Cellular Organization Within NB Cell-Laden Alginate Spheres

After 1 week of culture, cell-laden NB alginate spheres were processed for histological analysis. Samples were fixed in 4% paraformaldehyde, then paraffin embedded and sectioned (5 μm).

Deparaffinized and rehydrated paraffin sections were routinely stained with hematoxylin and eosin (H&E) and observed under optical microscope (Nikon h550L).

Other hydrated sections were processed for AFM analysis. In particular, re-hydrated samples were let dry in laminar flow box and analyzed by AFM (Nanowizard3, JPK, Berlin, Germany). Due to the adhesiveness of the sample, topography imaging of the embedded cells and surrounding gel were reconstructed using the point of contact of high-resolution (128*128 points) force-distance maps performed in air. A soft cantilever NITRA-TALL B (AppNano, Mountain View, CA, USA), with nominal spring constant 0.1N/m, was calibrated calculating the deflection sensitivity against a freshly peeled mica sheet and subsequently using the thermal noise method. Obtained images were post-processed in Gwyddion 2.50.

### NB Cell-Laden Alginate Spheres Viability, Proliferation, and Drug Sensitivity

NB cells viability within alginate-based tumor models was evaluated through a live/dead assay (Sigma Aldrich). Briefly, after 24 h NB cell-laden alginate spheres were washed with PBS and incubated in 2 mM calcein AM and in 4 mM EthD-1 in PBS for 15 min at 37°C in a dark environment, to detect live and dead cells, respectively. NB cell-laden alginate spheres were washed three times in PBS. Positivity to either staining solution was observed by means of fluorescence microscopy (Nikon H550L).

NB cells proliferation within tumor models was quantitatively assessed through Presto Blue Assay (Thermo Fisher Scientific). NB cells cultured over 24 wells were used as control. In brief, after 1 day (DAY 1), 3 days (DAY 3), and 7 days (DAY 7) of culture the medium was removed from all samples and replaced with fresh medium containing 1% v/v of Presto Blue solution, as indicated by the manufacturer. Samples were incubated at 37°C for 1.5 h in dark. The supernatants were collected and absorbance readings at 570 and 600 nm assessed spectrophotometrically.

For the analysis of drug sensitivity, HTLA-230 cell-laden alginate spheres were treated for 24 h with decreasing concentration of clinical-grade imatinib mesylate (Selleck Chemicals, USA). Drug cytotoxicity on NB cell lines was quantitatively assessed through Presto Blue Assay. Briefly, the medium was removed from samples and replaced with fresh medium containing 1% v/v of Presto Blue solution, and incubated at 37°C for 1.5 h in dark. The supernatants were collected and absorbance readings at 570 and 600 nm assessed spectrophotometrically. 3D results were compared with conventional 2D cultures.

### Immunostaining

NB-alginate spheres were cultured with and without INF-γ (PeproTech, Rock Hill, NJ) at the final concentration of 100 ng/ml. After 7 days, samples were washed three times with PBS and incubated with the appropriate primary mAb for 1 h at room temperature. After washing three times in PBS, samples were incubated for 1 h at room temperature with Phycoerythrin (PE)-conjugated isotype-specific goat anti-mouse second reagent (Southern Biotechnology Associated, Birmingham, AL). Imaging was performed with a fluorescence microscope (Nikon H550L). Quantification of fluorescence was carried out through Image J® software from fluorescent images. In particular, measurements of the positive stained area were assessed through a semi-automatic image post-processing of the binarized images. All the measurements were carried out in quintuplicate for each category of samples.

### Monoclonal Antibodies

The following primary mAbs were produced in our laboratories: M5A10 (IgG1, anti-PVR), L14 (IgG2a, anti-nectin-2), BAM195 (IgG1, anti-MICA), A6136 (IgM, anti-HLA class-I), NE97 (IgG2b, anti-B7-H3) (25), BAB281 (IgG1, anti-NKp46) (8). Anti-PD-L1.3.1 (IgG1, anti-PD-L1), and anti-PD-L2 (IgG1) were produced in D. Olive lab. D1.12 (IgG2a anti-HLA-II) mAb was produced by R. Accolla (University of Insubria, Varese, Italy). Anti-ULBP1-4 were purchased from R&D System (Minneapolis, US) and Santa Cruz Biotecnology (Dallas, US); anti-GD_2_ (IgG2a) was purchased from BD Bioscience PharMingen, San Diego, CA); anti-CXCR4 (IgG2b) was purchased from &D Systems (Minneapolis, MN).

The following fluorochrome-conjugated anti-KIR in combination with anti-CD3-PECF594 (IgG1; BD Bioscience) and anti-CD56-PC7 (IgG1; Beckman Coulter, Marseille, France) mAbs were used: GL-183 (IgG1, anti-KIR2DL2/L3/S2; Beckman Coulter), EB6B (IgG1, anti-KIR2DL1/S1 and KIR2DL3*005; Beckman Coulter), Z27 (IgG1, anti-KIR3DL1/S1; Beckman Coulter), DX9 (IgG1, anti KIR3DL1; Miltenyi Biotec, Bergisch Gladbach, Germany), FES172 (IgG2a, KIR2DS4; Beckman Coulter), 143211 (IgG1, anti-KIR2DL1 and KIR2DS5; R&D). ECM41 mAb (IgM, anti-KIR2DL3, with the exception of *005; Our laboratory) followed by FITC-conjugated goat anti-mouse IgM (Southern Biotech, Birmingham, AL) was also used.

### Immunofluorescence and Flow Cytometry

NB cells were detached by the use of the chelating agent EDTA (2D cultures) or recovered from 3D alginate hydrogels as described below. Cells were stained with the primary mAbs followed by Phycoerythrin (PE)-conjugated isotype-specific goat anti-mouse second reagents (Southern Biotechnology Associated, Birmingham, AL), and analyzed by flow cytometry (FACSCalibur Becton Dickinson & Co, Mountain View, CA). On every experimental session, the flow cytometer performances were monitored, the reproducibility of the fluorescence intensity was aligned by calibrated microsfere (Becton Dickinson & Co, Mountain View, CA) and isotype-matched irrelevant antibodies were used as controls.

Peripheral blood mononuclear cells (PBMC) were isolated by Ficoll-Hypaque density gradient from blood of healthy donors, upon informed consent, and frozen at −80°C. KIR surface expression by NK cells was analyzed on freshly derived or thawed PBMC by immunofluorescence. Appropriate combinations of fluorochrome-conjugated anti-KIR mAb were used to identify inhibitory and activating KIR. Cytofluorimetric analysis was performed by MACSQuant-analyzer (Miltenyi-Biotec, Bergisch Gladbach, Germany). Data were analyzed using FlowJo Version 10 (TreeStar).

### Recovery of NB From Alginate Hydrogels and CD107a Degranulation Assay

NB cell-laden hydrogels were treated with alginate solubilizing solution (0.15M NaCl and 55 mM Sodium Citrate, Sigma Aldrich) in a volume of 100 μl/hydrogel, incubated at 37°C until alginate fully dissolves (about 20 min), washed twice with complete medium and pelleted by centrifugation (1400 rpm/7 min).

In degranulation assays PBMC from KIR2DL1^pos^, KIR2DL2/S2^neg^, KIR2DL3^pos^, KIR3DL1^pos^ HLA-C1/C2 donors were used as effectors and HTLA-230 (KIR-L: HLA-A3, HLA-Bw4, HLA-C1/C1) recovered from alginate hydrogels as targets. PBMC were thawed, cultured for 3 days in the presence of IL-2 (600 IU/ml) to stimulate NK cell populations, incubated for 3 h with target cells in the presence of a PE-conjugated anti-human CD107a (LAMP-1) (IgG1; BD Biosciences) and analyzed by flow cytometry.

### RNA Extraction and Real Time PCR

NB cells were recovered from the alginate hydrogels as above described and total RNA was extracted by using the miRCURY RNA Isolation Kit—Cell and Plant (Exiqon). 250 ng of RNA were reverse transcribed using the SuperScript VILO cDNA Synthesis Kit (Invitrogen). The cDNA was amplified by real-time PCR using specific TaqMan Gene Expression Assays (Applied Biosystems). Expression of B7-H3, PD-L1, PD-L2, and PVR genes was normalized to the GAPDH gene expression. Experiments were performed in triplicate.

### Statistical Analysis

For the quantitative evaluation of the morphological parameters of NB cell-laden alginate spheres over time and proliferation rate, data were analyzed by Student's paired *t*-test and statistical significance was set at *p* < 0.05, *n* = 5.

Student's paired *t*-test was performed for drug sensitivity analysis and statistical significance was set at *p* < 0.05, *n* = 4.

For the quantitative analysis of the fluorescent images, measurements were analyzed by Student's paired *t*-test and the statistical significance (p) is indicated, *n* = 5.

Wilcoxon-Mann-Whiteny *p*-value test or Student's *t*-test were employed for cytofluorimetric analysis and real time PCR, respectively. The statistical significance (p) is indicated. Graphic representation and statistical analysis were performed using the PASW Statistic version 20.0 software (formerly SPSS Statistics) (IBM, Milan Italy) and GraphPad Prism 6 (GraphPad Software La Jolla, CA).

## Results

### NB Cell-Laden Hydrogel Tumor Model

**Figure 1:**
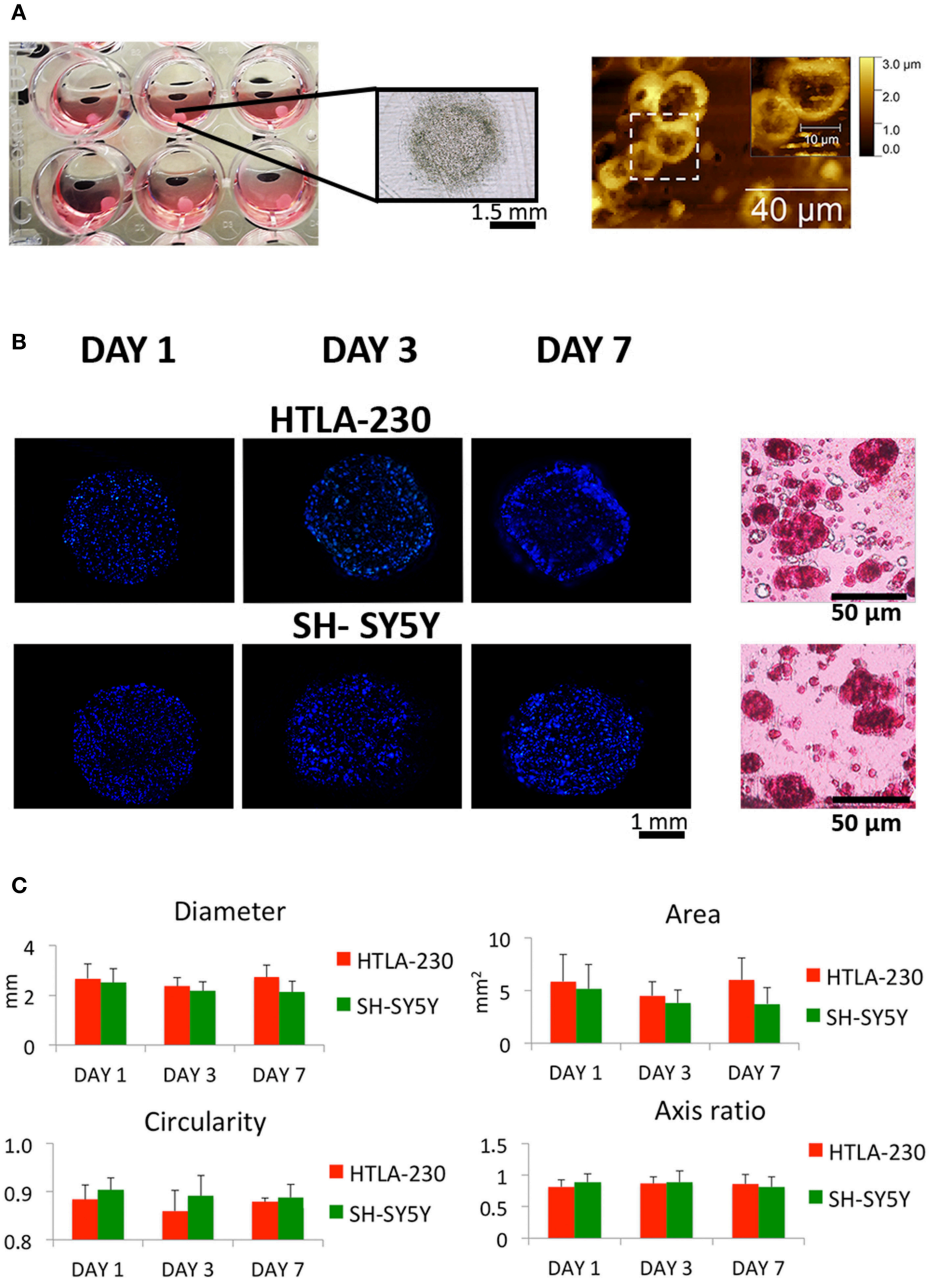
NB cell-laden hydrogel tumor model. **(A)** Picture, optical image of NB-loaded alginate spheres and AFM figures showing NB cells clusters within the hydrogels. **(B)** Representative images of DAPI-stained NB cells nuclei within alginate spheres at different time points and histological sections stained with H&E after 7 days of culture. **(C)** Quantitative analysis of the morphological parameters of the NB cell-laden alginate spheres to monitor the temporal change in the hydrogel dimension (mean ± SD) up to 1 week of culture.

We selected two prototypic NB cell lines, HTLA-230 characterized by MYCN amplification and 1p deletion, and SH-SY5Y that lacks MYCN amplification and 1p deletion (31). NB cells were embedded into alginate spheres, cultured for 1 week as described in materials and methods section, and the morphological properties analyzed at different time points (Figure 1).

NB cells grown within the 3D alginate spheres presented a spheroid morphology, high nucleus/cytoplasm ratio and clustered organization, resembling *in vivo* organization and phenotype, as shown in the histological and Atomic Force Microscopes (AFM) images (Figures 1A,B).

The NB cell-laden hydrogels had a spherical shape, which remained quite constant up to the later stage of culture (Figures 1B,C). Uniform and reproducible shape and size of *in vitro* tumor models are crucial for their potential use as relevant informative tools in therapies/drug testing (45). Therefore, a quantitative analysis over time of NB cell-laden hydrogels in terms of variations of diameter, area, circularity, and axis ratio (representative of the size and shape, respectively) was performed to assess their potential and translational value.

Several cell densities (2 × 10^6^ cells/mL; 4 × 10^6^ cells/mL; 8 × 10^6^ cells/mL) have been tested (data not shown) to guarantee a proper cell-cell contact within the hydrogel and, at the same time, to avoid cellular overgrowth with the subsequent loss of hydrogels spherical and regular conformation over time.

Interestingly, a relatively low cell density (2 × 10^6^ cells/mL) guaranteed regular hydrogel shape and size up to 1 week of culture (Figure 1C). This is in agreement with other studies, which reported that tumor models with low cell density are more homogeneous and regular if compared with self-aggregated tumor spheroids (45).

### NB Cell-Laden Alginate Spheres Viability, Proliferation, and Sensitivity to Imatinib Mesylate

High cellular viability and proliferation rate are key aspects in designing hydrogels as *in vitro* cancer models. After 24 h of culture, we tested NB cells viability by using the Calcein-Acetyoxymethyl ester (Calcein AM) viability assay, discriminating live and dead cells, as green or red fluorescent cells respectively.

**Figure 2:**
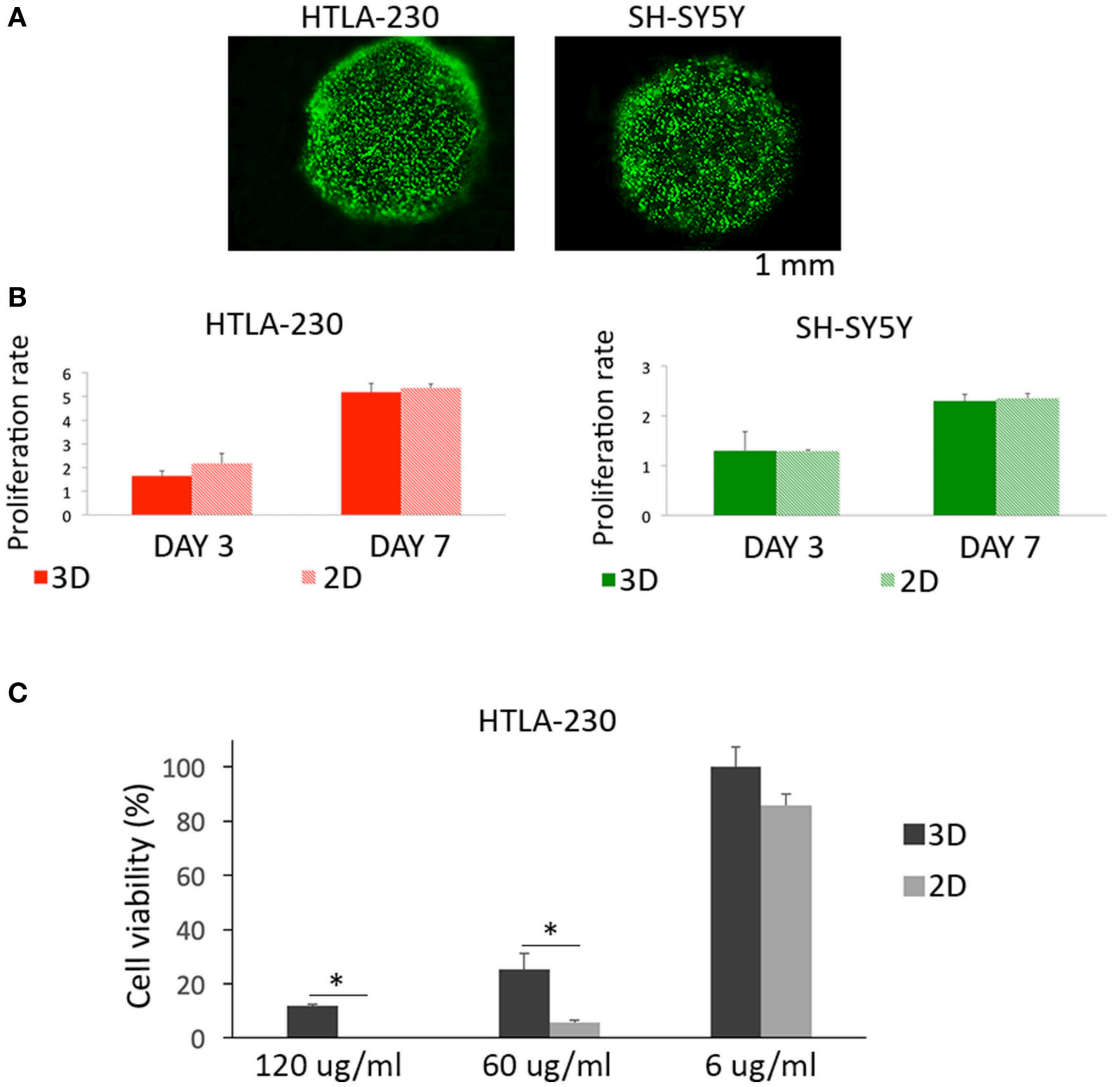
Cell viability, proliferation, and sensitivity to imatinib mesylate. **(A)** Cell viability represented by live/dead images of NB cell-laden alginate spheres after 24 h of culture. **(B)** Quantitative analysis of the proliferation rate of NB cells embedded within alginate spheres assessed through Presto Blue assay. **(C)** Cell viability of HTLA-230 cell-laden alginate spheres treated for 24 h with the indicated concentration of imatinib mesylate. Cell viability was derived as % of alive cells normalized respect the untreated controls. Values are reported as mean+S.E. *P < 0.05.

We observed, for both cell lines, a cell viability >95%, suggesting that the methodological approach needed for cells embedment within the alginate spheres is not harmful for NB cells (Figure 2A).

Moreover, we compared up to one-week alginate spheres and traditional 2D culture system for their capability of supporting NB cell proliferation (Figure 2B). This analysis showed a similar proliferation rate for NB cells expanded by the two different methods, proving that alginate spheres allow an optimal diffusion of nutrients and vital gases toward NB cells. Finally we compared the sensitivity of 3D and 2D cultured NB cells to the cytotoxic effect of imatinib mesylate (Figure 2C). This tyrosine kinase inhibitor provided benefits in some NB patients (46) and in 2D showed both on target and off target effects (47). As in 2D, the viability of NB cell-laden alginate spheres was not significantly affected by low drug concentrations. On the contrary, NB cell-laden alginate spheres were significantly more resistant than 2D-cultured tumor cells to the cytotoxic drug concentrations used. This suggests that 3D model may be a more suitable and reliable platform for testing drugs with respect to 2D standard cultures.

### Constitutive and IFN-γ-Induced Immunophenotype of NB Cell-Laden Alginate Spheres

In order to support the concept that NB cell-laden alginate spheres might represent a systematic and reproducible pre-clinical tool to test innovative NK-based immunotherapeutic approaches, we verified whether NB cells expanded within alginate spheres expressed the repertoire of ligands previously described in 2D culture systems (19, 25). Moreover, we also analyzed the effect of IFN-γ, which in 2D has been shown to modulate the expression of HLA-I and PD-Ls immune checkpoint molecules (31). NB cell-laden alginate spheres were stained with specific mAbs and imaging was performed with a fluorescence microscope, as described in materials and methods section.

**Figure 3:**
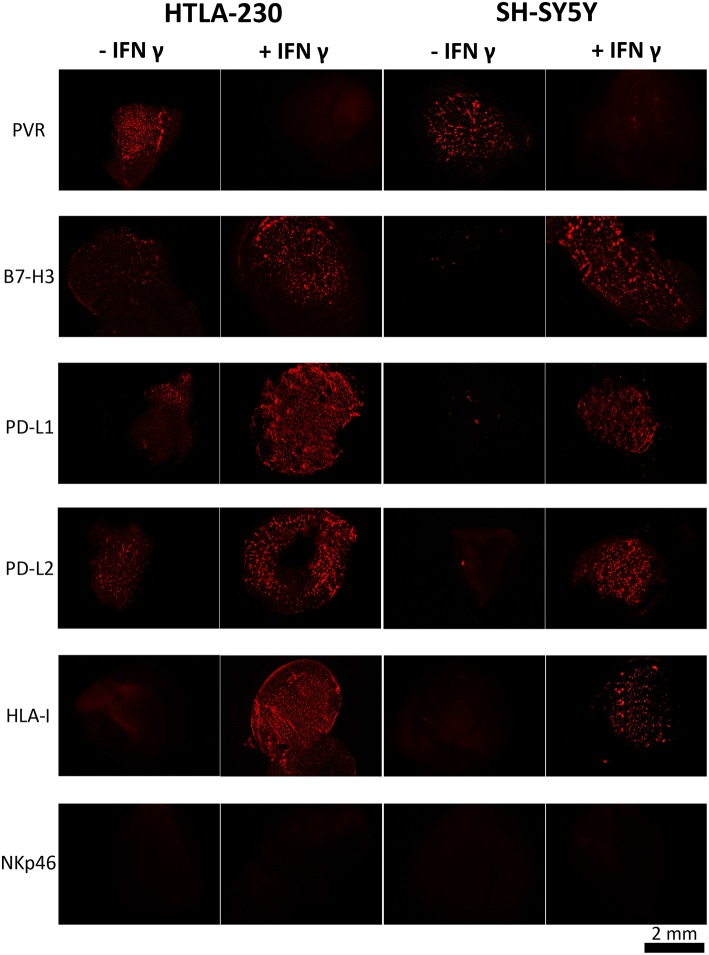
INF-γ effect in NB cells cultured within 3D alginate spheres. Immunofluorescent images of PVR, B7-H3, PD-L1, PD-L2, and HLA-I expression (in red) in NB cell-laden alginate spheres cultured a week either in the absence or in the presence of INF-γ. The anti-NKp46 mAb is used as a negative control.

HLA-I was undetectable in both NB cell lines. On the contrary, they were positive for PVR, a ligand of DNAM-1 activating receptor, whose expression is associated to high susceptibility to NK-mediated killing (19) (Figure 3, Figures S1, S2). HTLA-230 also expressed B7-H3, PD-L1, and PD-L2, whereas these molecules were virtually undetectable in SH-SY5Y cells (Figure 3).

Upon IFN-γ-treatment NB cell-laden alginate spheres *de novo* expressed HLA-I molecules. Moreover, upregulation (HTLA-230) or *de novo* expression (SH-SY5Y) of B7-H3, PD-L1, and PD-L2 immune checkpoint ligands was detected. Interestingly, these effects were accompanied by downregulation of PVR, which virtually disappeared (Figure 3 and Figure S2).

### Phenotypic and Functional Analysis of NB Cells Recovered From Alginate Hydrogels

**Figure 4:**
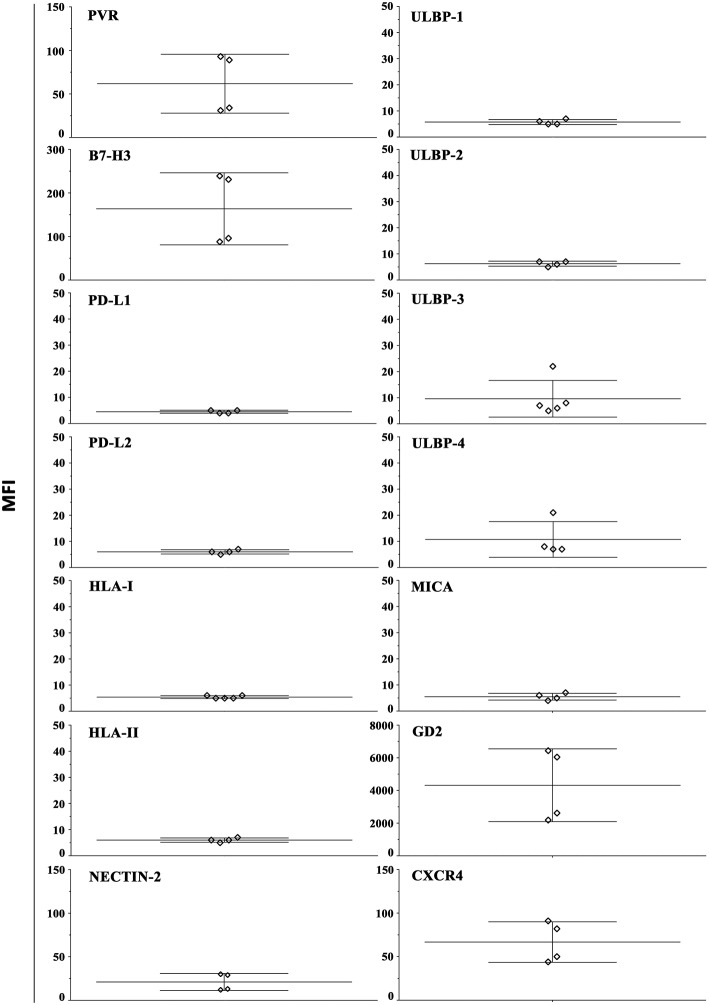
Constitutive surface phenotype of NB cells recovered from 3D alginate hydrogel. HTLA-230 cells recovered from alginate hydrogels were evaluated by immunofluorescence and flow cytometry for the expression of the indicated molecules. Four independent experiments are shown. The Median Intensity Fluorescence (MFI) is shown.

In order to evaluate whether our 3D model can allow recovering NB cells suitable for phenotypic and functional studies, HTLA-230 cells were gently extracted from alginate hydrogels (see materials and method). The constitutive expression of different surface molecules was analyzed by immunofluorescence and flow cytometry (Figure 4 and Figure S3A). With the exception of PD-L1, the analysis confirmed the ligand repertoire detected in NB cell-laden alginate sphere (Figure 3). Moreover, we could analyze other molecules regulating NK cell activity or associated with NB biology. We observed a consistent good expression of nectin-2 (an additional ligand of DNAM-1) (14), GD_2_ (a NB-associated antigen) (2) and of CXCR4 (48). Other molecules including HLA-II (interacting with the LAG-3 immune checkpoint receptor) and various NKG2D ligands (MICA, ULBP1-4) were virtually undetectable.

We also tested the capability of recovered NB cells, either untreated or treated with IFN-γ, to induce NK cells degranulation (Figure S3B, right). The efficacy of IFN-γ treatment was checked analyzing HLA-I expression (Figure S3B, left). Effectors were represented by NK cells from peripheral blood of healthy donors, who have been selected considering the HLA-I haplotype of HTLA-230 target cells. In particular, two donors were chosen that presented a subset of educated KIR/KIR-L mismatched NK cells, e.g., NK cells expressing inhibitory receptors specific for a KIR-L present in the donor and absent in target cells. Untreated (HLA-I^neg^) NB cells induced degranulation in both KIR/KIR-L mismatched and matched NK cell subsets. On the contrary, IFN-γ treated NB cells, that upon cytokine conditioning expressed protective amounts of HLA-I molecules, induced degranulation in KIR/KIR-L mismatched but not in KIR/KIR-L matched NK cells.

### Gene Expression Analysis of NB Cell-Laden Alginate Spheres

**Figure 5:**
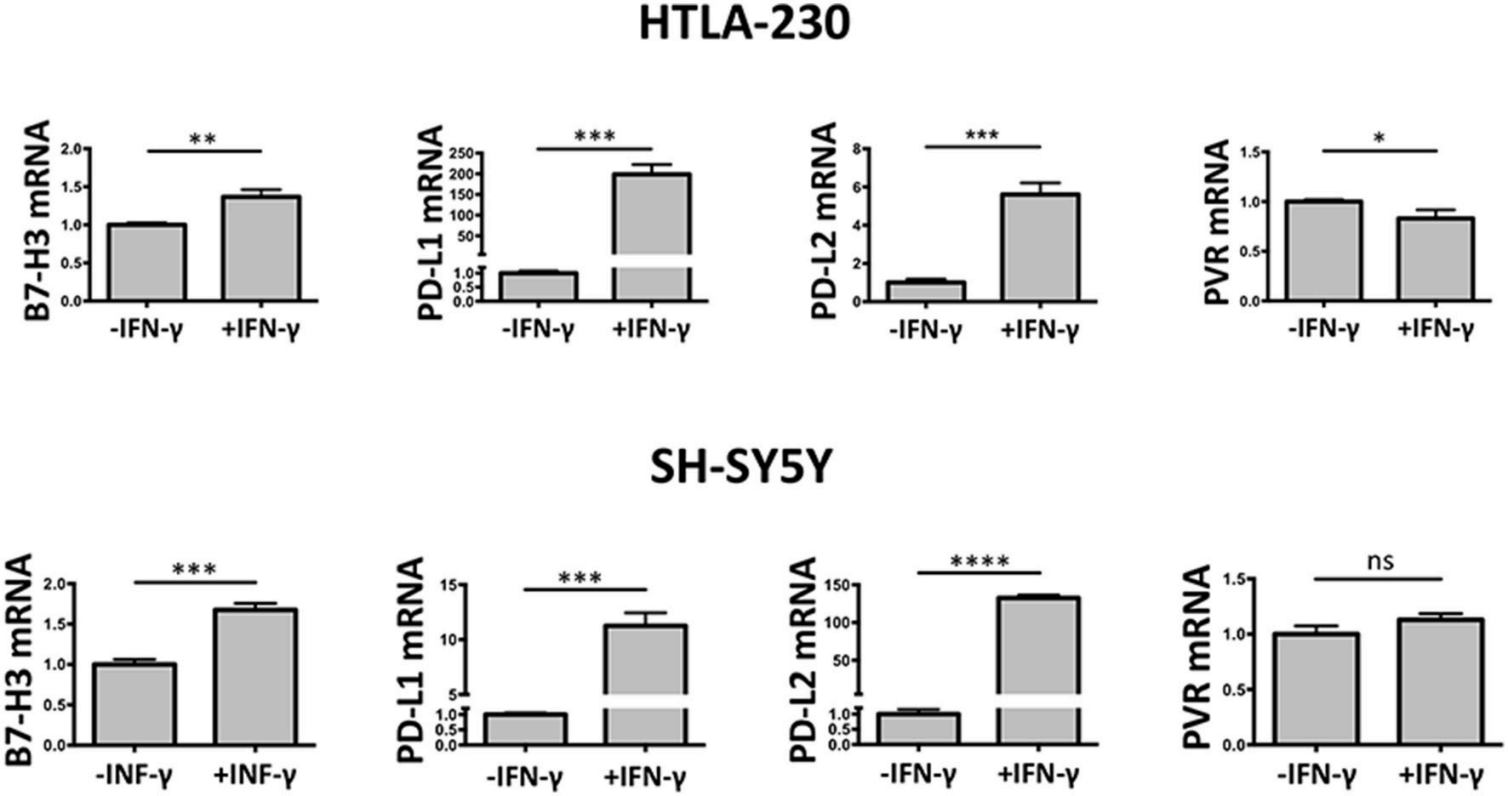
Real-time PCR analysis of NB cells recovered from 3D alginate hydrogel. Expression of target genes is normalized to that of the GAPDH reference gene. Data in triplicate are shown. Expression relative fold changes are referred to the gene expression in untreated cells, whose expression has been arbitrarily assigned the value 1. Student's *t*-test. ns, not significant; *P < 0.05; **P < 0.01, ***P < 0.001, ****P < 0.0001.

To verify at what level occurred the IFN-γ regulation of the immune checkpoint molecules and PVR ligand, 3D cultured HTLA-230 and SH-SY5Y cell lines were recovered at day 7, and analyzed by real-time PCR for the expression of the molecules of interest. As shown in Figure 5, in both cell lines IFN-γ treatment significantly increased the mRNA expression levels of B7-H3, PD-L1, and PD-L2. This was paralleled by decreased mRNA expression of PVR, which however was detectable in the HTLA-230 cell line only.

These data confirmed the results obtained by immunostaining of NB cell-laden alginate spheres and immunofluorescence analysis, and suggested that the IFN-γ-mediated modulatory effect occurred mainly at transcriptional level.

### Constitutive and IFN-γ-Induced Immunophenotype of NB Cultured in 2D Systems

Immunofluorescence analysis of NB cell-laden alginate spheres has been performed extending the cell cultures over a week, whereas previous published analyses carried out in a traditional 2D culture system were performed at earlier time points (31). Thus, in order to better compare NB cell-laden alginate spheres with conventional 2D cultures, HTLA-230 and SH-SY5Y NB cell lines have been cultured in 2D for 7 days, either in the absence or in the presence of IFN-γ, and their immunophenotype analyzed by immunofluorescence and flow cytometry.

**Figure 6:**
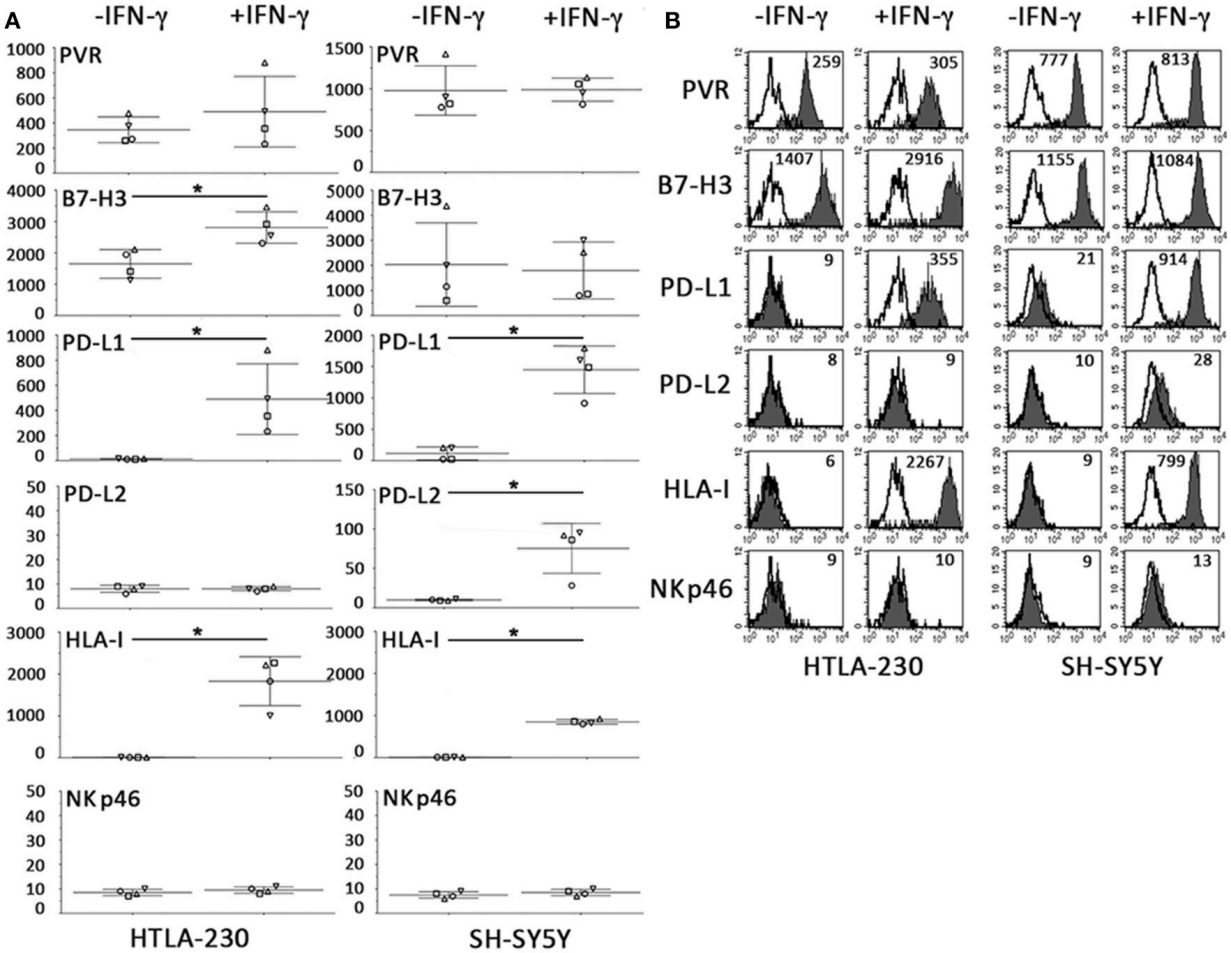
Constitutive and IFN-γ-induced immunophenotype of NB cells cultured for 7 days in 2D systems. **(A)** Surface phenotype of NB cell lines cultured a week either in the absence or in the presence of IFN-γ (immunofluorescence and flow cytometry). The anti-NKp46 mAb is used as a negative control. Four independent experiments with mean, 95% confidence intervals, and significance are shown. *p < 0.05. **(B)** A representative experiment is shown. White profiles refer to cells incubated with isotype-matched controls. Values inside each histogram indicate the MFI.

As shown in Figure 6, the constitutive immunophenotype of 2D NB cells only partially overlapped with that of NB cell-laden alginate spheres (see Figure 3). 2D NB cell were HLA-I^neg^ and PVR^pos^, but presented a different repertoire or surface densities of immune checkpoint molecules. In particular, both cell lines expressed high levels of B7-H3, and among the PD-Ls only PD-L1 was detected, whose expression was restricted to SH-SY5Y.

Relevant differences also existed upon IFN-γ treatment. As in 3D, IFN-γ increased the HLA-I and PD-L1 expression. On the other hand, in 2D system, increments in surface expression levels of B7-H3 and PD-L2 were only detected in 2D-cultured HTLA-230 and SH-SY5Y, respectively (Figure 6). Notably, the strong downregulation of PVR observed in IFN-γ-treated NB cell-laden alginate spheres, was not observed in 2D NB cells even after 7 days of culture.

## Discussion

In the last decades 2D cultures significantly contributed to the identification and characterization of molecules involved in anti-tumor immune responses. However, it is known that culturing cancer cells in a 3D niche may better reproduce the tumor architecture respect standard 2D cultures. 3D structures often significantly increase malignant phenotypes of cancer cells, which, instead, are suppressed in cells cultured in 2D conditions (49). This can explain the lower sensitivity to chemotherapeutic agents observed in 3D culture conditions as compared to 2D systems (50, 51).

Here we developed a novel 3D alginate-based hydrogel for culturing NB cells and evaluated the effects of the three-dimensionality on different NB cell properties such as morphology, cell viability, proliferation, sensitivity to drugs and immunophenotype. The novelty of our model relies on the fact that the few alternative 3D hydrogels proposed as NB models are based on natural polymer like collagen and Matrigel (50, 52–54). However, polymers of animal origin often show some limitations as difficult reproducibility due to variations between the different lots and the scarce possibility of modulating the biomechanical features of the final hydrogel. On the contrary, alginate properties can be easily tuned for biofabrication specific needs in order to obtain 3D chemically stable structures with optimal features.

In addition, the goal of the work was to develop a 3D hydrogel to evaluate the effects of the three-dimensionality on different NB cell features. Alginate is an inert polymer, differently from collagen and protein based natural polymers, because it lacks the native bonds that are usually responsible of the interactions with mammalian cells. Therefore, the choice of this polymer allowed to better decouple the contribution of the three-dimensionality on cell fate from a possible chemical conditioning due to the bioactivity of the substrate. However, most of the current 3D models for neuroblastoma are based on growing cells as spheroids starting from single cell suspensions (55–58). Although they replicate specific features of *in vivo* tumors including central hypoxic conditions, heterogeneity in phenotype and gene expression and altered cellular metabolism, the tumor spheroid model suffers from some limitations, particularly due to a lack of surrounding extracellular matrix components. This hampers the possibility to add other cell types, different from tumor cells with the aim to reproduce the cellular complexity of tumor microenvironment.

An additional novelty in our study is represented by the kind of analysis that has been performed. Indeed for the first time to our knowledge, we analyzed the expression in a 3D *in vitro* system of a wide repertoire of surface ligands crucial in the regulation of NK- and T- mediated immune responses against tumors.

As first result, we found that two prototypic NB cells (HTLA-230 and SH-SY5Y) differing for the amplification status of the prognostic factor MYCN (31), proliferated within our hydrogel spheres with a rate comparable with traditional 2D cultures, but with a spheroid morphology and a clustered organization, resembling the *in vivo* phenotype (59), revealing the suitability of the alginate-based model.

It is known from the literature that matrix stiffness plays an important role in modulating *in vitro* tumors progression (60, 61). For this reason, the polymer concentration was properly set based on our previous work (42), which claimed that, among hydrogels with different concentrations (0.5% w/V, 1%w/V, 2%w/V), the lowest concentrated alginate-based hydrogel was the best in supporting breast cancer cell lines proliferation *in vitro*. Hydrogels with a concentration lower than 0.5% w/V did not possess a proper robustness and, therefore, couldn't guarantee a structural stability during the period of *in vitro* cell culture.

Specifically, Marrella et al. reported that when cells are cultured over artificial environments their preference toward a substrate depends on the similarity of physical-mechanical proprieties of the substrate with ones of the cell native tissue (62). Therefore, soft substrates represent proper environments where to grow cells derived from soft tissues, such as breast, or nervous tissue.

These considerations are consistent with previous reports (63), where the increasing of matrix stiffness of collagen-based 3D hydrogels led to a decreased proliferation of glioma cells. In fact, brain tissue is one of the softest tissues in the human body (64) and extensive *in vitro* studies have shown that neurons, glia and neural stem/progenitor cell better respond when are cultured over soft substrates (65–69). Thus, due to the similarities between NB cells and the cells of embryonic neural crests from which NB derives, our culture system was based on a soft 3D alginate-based model.

Besides supporting NB cells proliferation, this model appeared to allow an optimal diffusion of nutrients and vital gases as revealed by the high viability of NB cells encapsulated within the hydrogels. Moreover, it preserves the proper physiology of NB cells growing in tumor microenvironment *in vivo* as also indicated by the higher drug resistance of NB cell-laden alginate spheres as compared to 2D systems. The effective diffusion of soluble molecules in our 3D alginate-based model was confirmed by the effect in NB cell-laden alginate spheres of IFN-γ, a crucial immunostimulatory cytokine released by activated NK and T helper lymphocytes. IFN-γ promotes the activity of different immune cell types including DC and macrophages that, acquiring high levels of HLA-I and HLA-II molecules, optimize antigen presentation to T lymphocytes. Importantly, immune responses need to be modulated in their duration and amplitude in order to achieve their resolution, avoiding the establishment of autoimmune aggression. The mechanisms preventing such detrimental events include the expression of immune checkpoint ligands such as PD-L1 and PD-L2 (23) acquired by Antigen Presenting Cells and by other cell types during the late phase of the immune responses. Importantly, it has been shown in 2D cultured systems that IFN-γ increased PD-Ls surface expression not only in healthy cells but also in different tumors, including NB (70), which become protected from the NK (and T)-mediated attack. IFN-γ-conditioning also led to the *de novo* acquisition of high levels of HLA-I, as demonstrated in NB cell lines and bone-marrow NB metastasis (31). HLA-I expression may exert both positive and negative effects, rendering NB cells more susceptible to T cell-mediated recognition, but highly resistant to the NK-mediated attack.

Our 3D culture system confirms data obtained in conventional 2D, including the IFN-γ-mediated expression in NB cells of HLA-I and PD-Ls molecules. It is of note that phenotypic differences have been appreciated in NB cells cultured in 2D system for 48 h (31) or for a week (Figure 6). In particular, the IFN-γ-mediated upregulation of B7-H3 in HTLA-230 cell line was appreciated only in cells cultured for 7 days. Moreover, differently from previous published results (31), low or null constitutive levels of PD-Ls and HLA-I were detected in 1 week cultured SH-SY5Y cells. This immunophenotype is more similar to that observed in SH-SY5Y cells xenografted in mice and removed after 3 weeks of growth. This suggests that in 2D culture systems variables may exist, including the number of cultured cells and cell-to cell interactions, that could impact on the surface expression of relevant molecules.

The 3D culture system also highlighted some unappreciated NB biological features. Among these, IFN-γ mediated an increment of the “NK-protective” ligands B7-H3 (25, 28, 71), PD-Ls and HLA-I that was higher in MYCN amplified than in non-amplified NB cells. This observation is particularly relevant if considering that MYCN amplified NBs represent the most aggressive subtype, often resistant to combined therapies. Standard therapy for high-risk NB patients includes an immunotherapy approach based on the use of antibodies targeting the disialoganglioside GD_2_, a tumor-associated antigen expressed by most NBs (72). In this context, NB cell-laden alginate spheres maintain a high expression of GD_2_. The efficacy of antibodies-mediated therapies relies on the triggering of FcγRs+ cells such as NK cells and macrophages that altogether release a great amount of IFN-γ, TNF-α, IL-1, and IL-6. Blood accumulation of these cytokines unleash a Cytokine Release syndrome (CRS), which represents a detrimental event also associated to therapies utilizing Chimeric Antigen Receptors (CAR)-engineered T and NK cells (73–75).

Notably, our 3D model showed that IFN-γ is also capable of reducing the expression of PVR, a ligand of DNAM-1, an activating receptor involved in NK-mediated recognition of various tumors including NB (16–18, 76, 77). Importantly, bone marrow metastasis lacking PVR expression have been detected in NB patients, suggesting that loss of PVR might occur *in vivo*, possibly potentiated by the pressure of immune system (25). Thus, PVR expression in NB cell-laden alginate spheres and its reduction upon IFN-γ-conditioning, phenomenon unappreciated in 2D standard culture conditions, well resemble the PVR variations occurring *in vivo*. This further suggests that NB cell-laden alginate spheres might represent a more appropriate culture system where to test immunotherapies. In particular, recent therapeutic approaches aim to target PVR, which is expressed by several tumor histotypes and by tumor-associated endothelium (14, 70). Strategies include PVR targeting using a modified attenuated poliovirus or T cell engineered with DNAM-1 chimeric antigen receptor (78–80).

Thus, the here proposed 3D model based on NB cell-laden alginate spheres appears to well preserve the physiology and immunophenotype of NB cells and might represent a reproducible and standardized tool to test different therapeutic approaches allowing a more precise monitoring of tumor responses as compared to animal preclinical models, often allowing tumor analysis only at few critical time points. Moreover, in our 3D model several test replicates can be easily performed, ensuring a faster achievement of statistical significance of the experiment with reduction of costs and animal sacrifice in accordance with 3R European rules.

To conclude, 3D hydrogel cultures well resembled *in vivo* NB cells growth due to their spheroid morphology and highlighted a novel and clinical-relevant constitutive and IFN-γ-inducible NB immunophenotype. Our data suggest that an alginate-based *in vitro* culture model might be envisaged as a future platform where to culture patients derived tumor cells. This will be the next mandatory step of our 3D culture system approach, crucial for set up and validate personalized therapeutic approaches and for the evaluation of their efficacy.

## Data Availability

All datasets generated for this study are included in the manuscript and/or the Supplementary Files.

## Author Contributions

AM and AD performed most of the experiments and contributed to planning the work, to the interpretation of data, and to writing the paper. MA and GC provided bioengineering information useful to set the 3D model. BC contributed to performing the experiments. DO revised the paper. SR performed the quantitative PCR analysis and contributed to the interpretation of data and to writing the paper. CB contributed to planning the work, to the interpretation of data, and to writing the paper. RC and SS planned the work, contributed to the interpretation of data, and wrote the paper. DP and RM selected the NK cell donors, contributed to the interpretation of data, and to writing the paper.

### Conflict of Interest Statement

DO is cofounder and shareholder of Imcheck Therapeutics. SS and MA are cofounders and shareholders of React4life S.r.l. The remaining authors declare that the research was conducted in the absence of any commercial or financial relationships that could be construed as a potential conflict of interest.
