# Supplementary material for: Cell-Laden Hydrogel as a Clinical-Relevant 3D Model for Analyzing Neuroblastoma Growth, Immunophenotype, and Susceptibility to Therapies
Source: Front Immunol. 2019 Aug 9;10:1876. doi: 10.3389/fimmu.2019.01876 (PMC6697063; doi:10.3389/fimmu.2019.01876)
Supplement: Supplementary file 1 [file Data_Sheet_1.pdf]

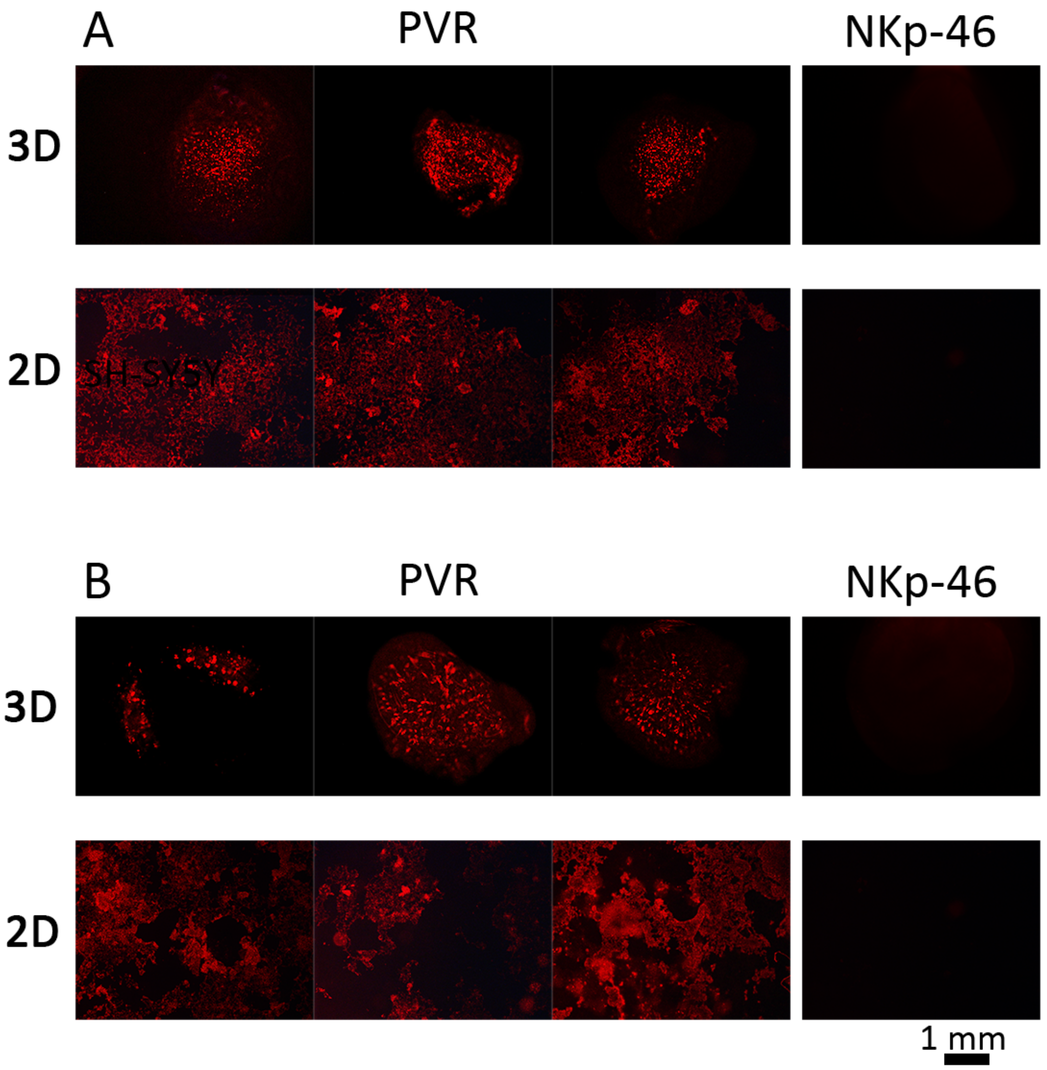

Figure S1. PVR expression of NB cells cultured within 3D alginate spheres and in 2D conditions. Immunofluorescent images representing PVR expression (in red) of HTLA-230 (A) and SH-SY5Y cells (B). The anti-NKp46 mAb was used as negative control.

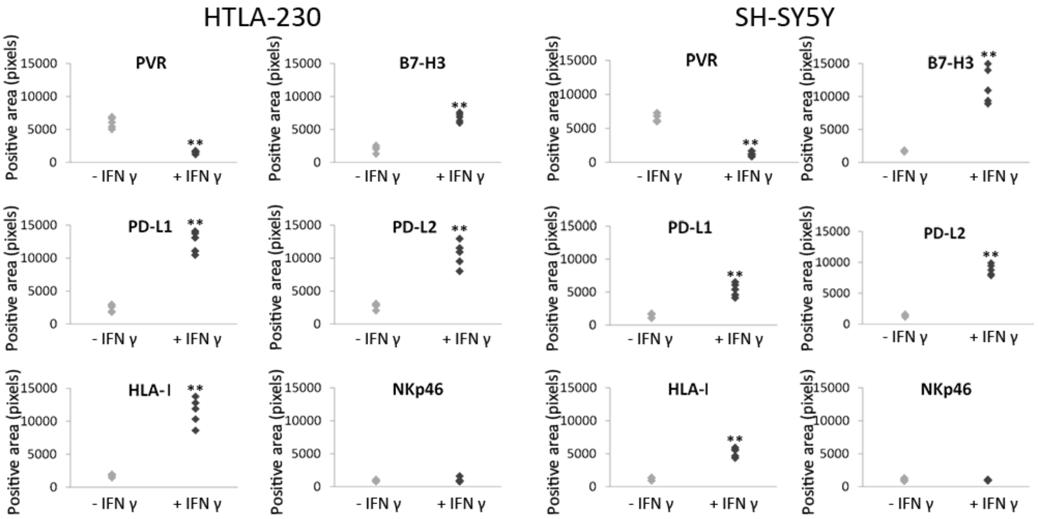

Figure S2. Quantitative analysis of the immunostaining intensity of PVR, B7-H3, PD-L1, PD-L2, HLA-I expression in NB cell-laden alginate hydrogels.

A quantification of the immunostaining intensity was performed to evaluate the expression of the ligands in NB cell-laden alginate spheres shown in Figure 3, either in the absence or in the presence of IFN-γ. All the measurements were carried out in quintuplicate for each category of samples. The anti-NKp46 mAb was used as negative control. Student's t-test. **: P<0,01.

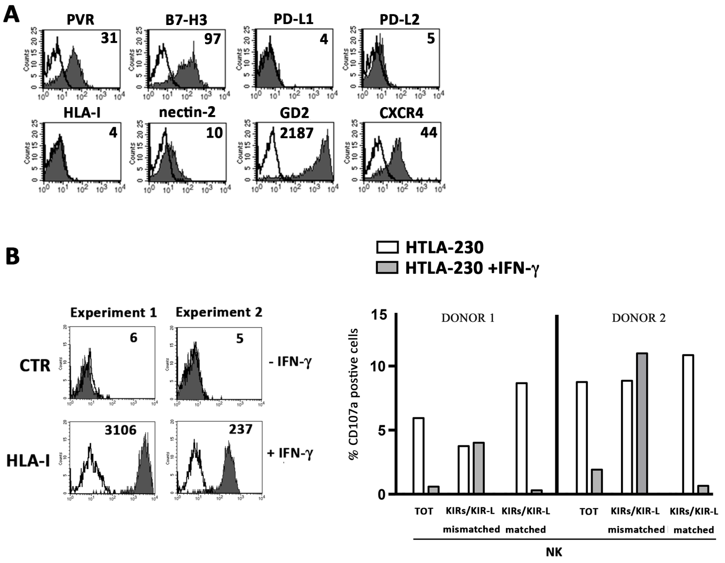

Fig.S3. Phenotypic and functional analysis of NB cells recovered from 3D alginate hydrogel.

(A) Surface phenotype of HTLA-230 cells recovered from 3D alginate hydrogel (immunofluorescence and flow cytometry). White profiles refer to cells incubated with isotype-matched controls. Values inside each histogram indicate the mean fluorescence intensity (MFI). (B) HTLA-230 cells, recovered from 3D alginate hydrogel untreated or treated with IFN-γ, were analyzed for HLA-I expression (left) and for the ability to induce NK cells degranulation (right). White profiles refer to cells incubated with isotype-matched controls. Values inside each histogram indicate the mean fluorescence intensity (MFI). The percentage of CD107a positive cells, normalized respect the spontaneous degranulation, has been analyzed gating on $CD56^{pos}CD3^{neg}$ cells (Total NK cells, TOT); $CD56^{pos}CD3^{neg}$ $KIR2DL1^{pos}KIR2DL3^{neg}KIR3DL1^{neg}$ $KIR3DL2^{neg}$ cells (KIR/KIR-L mismatched NK cell subset); $CD56^{pos}CD3^{neg}$ $KIR2DL1^{neg}KIR2DL3^{pos}KIR3DL1^{pos}KIR3DL2^{pos}$ cells (KIR/KIR-L matched NK cell subset).
